# Phosphodiesterase Type-5 Inhibitor Tadalafil Modulates Steroid Hormones Signaling in a Prostate Cancer Cell Line

**DOI:** 10.3390/ijms22020754

**Published:** 2021-01-13

**Authors:** Viviana M. Bimonte, Francesco Marampon, Ambra Antonioni, Simona Fittipaldi, Elisabetta Ferretti, Richard G. Pestell, Mariaignazia Curreli, Andrea Lenzi, Giovanni Vitale, Antonio Brunetti, Silvia Migliaccio, Antonio Aversa

**Affiliations:** 1 Department of Movement, Human and Health Sciences, “Foro Italico” University, 00135 Rome, Italy; v.bimonte@studenti.uniroma4.it (V.M.B.); silvia.migliaccio@uniroma4.it (S.M.); 2 Department of Experimental Medicine, “Sapienza” University of Rome, 00161 Rome, Italy; ambra.anto@hotmail.it (A.A.); elisabetta.ferretti@uniroma1.it (E.F.); mariaignazia.curreli@uniroma1.it (M.C.); andrea.lenzi@uniroma1.it (A.L.); 3 Department of Experimental and Clinical Medicine, Magna Græcia University, 88100 Catanzaro, Italy; 4 Department of Radiological, Oncological and Pathological Sciences, “Sapienza” University, 00161 Rome, Italy; francesco.marampon@uniroma1.it; 5 Department of Biomedicine and Prevention, “Tor Vergata” University, 00133 Rome, Italy; fttsmn01@uniroma2.it; 6 Pennsylvania Cancer and Regenerative Medicine Research Center, Baruch S. Blumberg Institute, Pennsylvania Biotechnology Center, Wynnewood, PA 19111, USA; richard.pestell@bblumberg.org; 7 Department of Medical Biotechnologies and Translational Medicine, University of Milan, 20122 Milan, Italy; giovanni.vitale@unimi.it; 8 Laboratory of Geriatric and Oncologic Neuroendocrinology Research, Istituto Auxologico Italiano, IRCCS, Cusano Milanino, 20095 Milan, Italy; 9 Department of Health Sciences, Magna Graecia University, 88100 Catanzaro, Italy; brunetti@unicz.it

**Keywords:** tadalafil, prostate cancer, androgen resistance, aromatase, bicalutamide

## Abstract

Background: The androgen receptor (AR) plays a key role in normal prostate homeostasis and in prostate cancer (PCa) development, while the role of aromatase (Cyp19a1) is still unclear. We evaluated the effects of a treatment with Tadalafil (TAD) on both these proteins. Methods: Androgen-sensitive human PCa cell line (LnCAP) was incubated with/without TAD (10^−6^ M) and bicalutamide (BCT) (10^−4^ M) to evaluate a potential modulation on cell proliferation, protein and mRNA expression of Cyp19a, AR and estrogen receptor-β (ERβ), respectively. Results: TAD increased early AR nuclear translocation (*p* < 0.05, after 15 min of exposure), and increased AR transcriptional activity (*p* < 0.05) and protein expression (*p* < 0.05) after 24 h. Moreover, after 24 h this treatment upregulated Cyp19a1 and ERβ mRNA (*p* < 0.05 and *p* < 0.005 respectively) and led to an increase in protein expression of both after 48 h (*p* < 0.05). Interestingly, TAD counteracted Cyp19a1 stimulation induced by BCT (*p* < 0.05) but did not alter the effect induced by BCT on the AR protein expression. Conclusion: We demonstrate for the first time that TAD can significantly modulate AR expression and activity, Cyp19a1 and ERβ expression in PCa cells, suggesting a specific effect of these proteins. In addition, TAD potentiates the antiproliferative activity of BCT, opening a new clinical scenario in the treatment of PCa.

## 1. Introduction

Prostate cancer (PCa) is the leading cause of male cancer death in western countries with an increasing incidence in developing countries [1]. Testosterone (T) or dihydrotestosterone (DHT) stimulate PCa cell growth by binding the androgen receptor (AR) [2]. Upon binding to the AR, the androgen/AR complex translocates to the nucleus where it dimerizes and binds to specific androgen responsive elements (AREs) within target gene promoters, leading to modulation of gene transcription [3]. Due to the importance of androgenic signal transduction, the androgen deprivation therapy (ADT) is widely used in clinical practices as adjuvant or neoadjuvant therapy, either alone or in combination with surgery and/or radiotherapy to block or slow down prostate cancer growth [4,5]. Nevertheless, ADT is invariably followed by the recurrence of castration-resistant prostate cancer (CRPC) with a disease relapse time within 12–18 months [6,7]. These events occur even earlier by repressing AR expression with androgen deprivation [8] and are facilitated by ADT [9]. Further, this event can evolve later by the inappropriate restoration of the AR signaling axis and the increased intracrine steroidogenesis [10]. Thus, in order to counteract and/or delay the onset of the CRPC phenotype, it may be important to find new therapeutic strategies able to support/restore AR expression at the earliest stages of disease.

Cyclic adenosine monophosphate (cAMP) and cyclic guanosine monophosphate (cGMP) are ubiquitous second messengers, which regulate multiple functions in virtually all eukaryotic cells. The deregulation of these factors has been involved in several pathophysiological processes and diseases, including cancer [11]. The phosphodiesterase (PDE) enzyme superfamily consists of 11 isoforms (PDE1–PDE11) that modulate the intracellular concentrations of cAMP and cGMP by catalyzing their degradation to inactive 5′ nucleotide monophosphates. Consequently, PDEs regulate many physiological processes whilst their altered expression, localization and function are implicated in the pathogenesis of several diseases [12,13], where these enzymes have been shown to be often over expressed and/or aberrantly activated, thus promoting the onset and progression of tumors [14]. In particular, increased expression of PDE5 has been reported in several human cancers [15,16,17,18], whereas its inhibition has been shown to induce anticancer effects [19,20,21]. These findings have been also observed in PCa, both in preclinical [22,23,24,25,26] and clinical experiences [27,28]. However, the molecular mechanism(s) by which PDE5 inhibition affects PCa homeostasis are unknown.

Interestingly, recent in vivo data suggest that estrogens may play a key role in prostate disease. Indeed, it has been reported that high estrogen levels can induce premalignant dysplasia and that their combination with high androgen levels may give rise to malignancy [29,30]. The production of estrogens from androgens is mediated by aromatase (Cyp19a1). This enzyme plays a pivotal role in cancer development in several tissues, particularly in the breast tissue, although its role and contribution in prostate carcinogenesis remains undefined [29,30]. Notably, PDE5 inhibition has a direct effect on the Cyp19a1 [31,32]. In vitro, in human osteoblastic cells, tadalafil (TAD), a PDE5 inhibitor (PDE5i), decreases Cyp19a1 expression and increases AR protein expression, suggesting a novel control of the steroid hormone pathway by PDE5i [32]. Moreover, TAD increases AR protein expression in C2C12 murine muscle cells, accelerating myogenic differentiation [33].

The aims of the present study were to investigate the effects of TAD on the expression of AR and Cyp19a1, and its potential impact in modulating the antiproliferative activity of ADT in human PCa LnCAP cells.

## 2. Results

### 2.1. Tadalafil Increases AR Expression and Function without Affecting LNCaP Cell Viability and the Proliferation Rate

**Figure 1:**
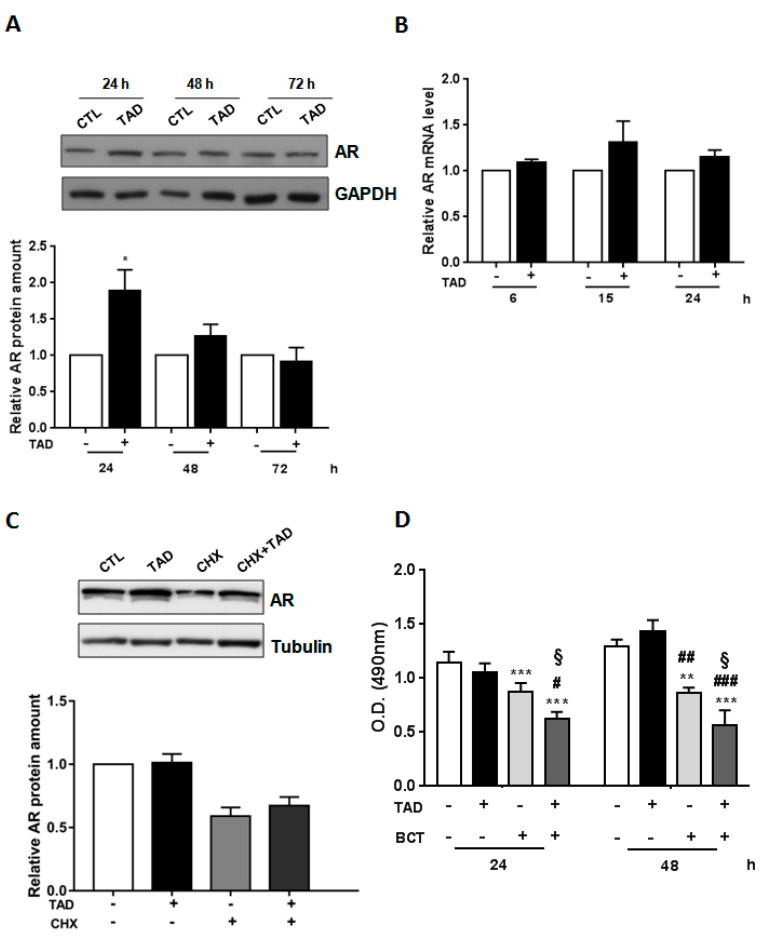
Effects of TAD on AR expression and LNCaP proliferation. (**A**) Representative Western blot analysis of AR protein expression in LNCaP cells cultured in the presence (+) or in the absence (−) of TAD (10^−6^ M) for 24, 48 and 72 h. GAPDH was used as an internal control for protein loading. (**B**) Effect of TAD on AR mRNA expression in the presence (+) or in the absence (-) of TAD (10^−6^ M). (**C**) CHX effect on AR protein expression. A representative Western blot of AR protein expression is shown in LNCaP cells cultured for 3 h in the presence (+) or in the absence (−) of TAD (10^−6^ M), and pretreated or not with CHX (10 µg/mL) for 1 hr. Tubulin, control for protein loading. (**D**) MTS proliferation assay in LNCaP cells in cultured cells either in the presence (+) or in the absence (−) of TAD (10^−6^ M) and/or bicalutamide (BCT, 10^−4^ M) for and 24–48 h. Results are represented as mean ± SE (*n* = 3) of three independent experiments. * *p* < 0.05, ** *p* < 0.005, *** *p* < 0.001 vs. CTL cells, # *p* < 0.05, ## *p* < 0.005, ### *p* < 0.001 vs. TAD treated cells, § *p* < 0.05 vs. BCT treated cells.

To evaluate the potential effects of TAD on AR expression and activity, LNCaP cells were incubated with TAD (10^−6^ M) for increasing time intervals (24, 48 and 72 h). As shown in Figure 1A, TAD significantly increased AR protein expression (24 h, TAD vs. CTL, *p* < 0.05), which returned to near baseline levels after 48–72 h (Figure 1A). The following question was whether TAD increased AR protein expression through transcriptional and/or translational mechanism(s). After 6, 15 and 24 h of incubation with TAD, no significant differences were observed in AR mRNA levels between untreated and treated cells (Figure 1B, TAD+ vs. TAD−). Since the half-life of AR protein in LNCaP cells, cultured in the absence of androgens, is approximately 3 h [34], we investigated whether TAD increased AR by a process of protein stabilization. To address this point, LNCaP cells were treated with cycloheximide (CHX 10 µg/mL), a well-known inhibitor of mRNA translation [35], in the presence or absence of TAD (10^−6^ M) for 3 h (Figure 1C). As shown in Figure 1C, TAD did not counteract CHX-induced AR protein downregulation (Figure 1C). Notably, since the treatment of LNCaP cells with TAD did not affect neither cell viability, nor cell proliferation rate at 24–48 h (Figure 1D), this is consonant with a non-transcriptional and non-translational effect of TAD on the upregulation of AR expression.

### 2.2. Tadalafil Promotes AR Nuclear Translocation and Transcriptional Activity

**Figure 2:**
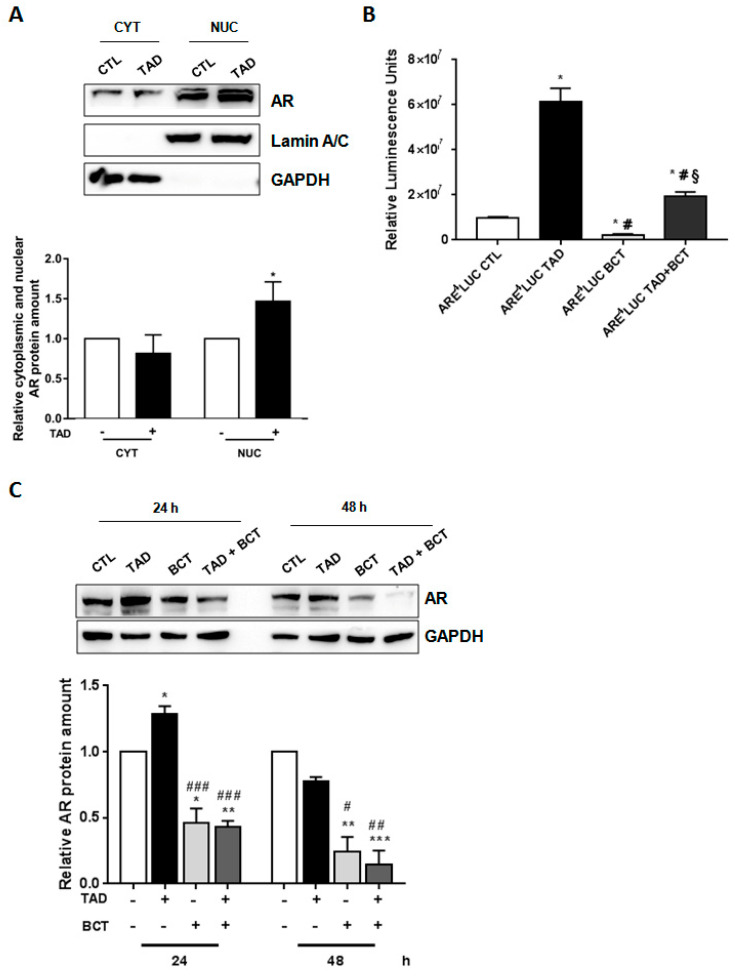
TAD boosts AR function and increases BCT-mediated AR downregulation. (**A**) Analysis of cytoplasmic (CYT) and nuclear (NUC) fractions of AR in LNCaP cells cultured in the presence (+) or in the absence (−) of TAD (10^−6^ M) for 15 min. GAPDH and Lamin A/C were used as loading and purity controls of each cellular fraction. (**B**) Effects of TAD on AR transcriptional activity. LNCaP were transfected with ARE^4^LUC and treated in the presence (+) or in the absence (−) of TAD (10^−6^ M) and BCT (10^−4^ M). Luciferase activity was measured after 24 h. (**C**) Representative Western blot analysis of AR protein expression in cultured cells either in the presence (+) or in the absence (−) of TAD (10^−6^ M) and/or BCT (10^−4^ M) for 24–48 h. In Western blot, GAPDH was used as a control of protein loading. Results are represented as mean ± SE (*n* = 3) of three separate experiments. * *p* < 0.05, ** *p* < 0.005, *** *p* < 0.001 vs. CTL cells, # *p*< 0.05, ## *p*< 0.005, ### *p*< 0.001 vs. TAD treated cells, § *p* < 0.05 vs. BCT treated cells.

LNCaP cells were used to verify whether TAD could affect AR translocation from cytoplasmic to nuclear compartment [36], and the transcriptional activity of AR. As shown in Figure 2A, 15 min of TAD (10^−6^ M) exposure significantly increased nuclear AR abundance (Figure 2A, TAD+ vs. TAD−, *p* < 0.05). Results were confirmed by the use of a known AR nuclear transport inhibitor, MDV3100 (MDV 10^−5^ M) [37]. In fact, LNCaP cells, treated with MDV (10^−5^ M) + TAD (10^−6^ M) for 15 min, showed a significant decrease of AR nuclear translocation (Figure S1, *p* < 0.05 vs. CTL cells, *p* < 0.05 vs. TAD treated cells, § vs. MDV treated cells).

To test AR transcriptional activity, LNCaP cells were transiently transfected with androgen-responsive luciferase reporter vectors, in which androgen-cis elements (ARE-Luc) were cloned upstream the luciferase gene, and treated with TAD (10^−6^ M) for 24 h, either in the presence or absence of the AR antagonist BCT (10^−4^ M). As shown in Figure 2B, TAD significantly increased luciferase activity (Figure 2B, ARE^4^LUC TAD vs. ARE^4^LUC CTL, *p* < 0.05) and efficiently counteracted luciferase activity inhibition induced by BCT (Figure 2B, ARE^4^LUC TAD + BCT vs. ARE^4^LUC CTL + BCT, *p* < 0.05), strongly suggesting an AR dependent effect. By contrast, luciferase assay, performed in DU145 and PC3 cells (androgen-insensitive cells lines), showed that exposure to TAD and TAD + BCT did not affect AR activity (Figure S2A). Additionally, TAD treatment induced an increase of AR downstream target gene (Figure S2B). Indeed, long-term TAD exposure (72 h) significantly increased PSA mRNA level, (Figure S2B, *p* < 0.05 vs. CTL cells). Cells were then exposed to BCT, which inhibited the PSA production in LNACap cells [38,39]. Interestingly it was observed a reduction of PSA in cells treated with either BCT alone or BCT + TAD (Supplementary Figure S2B, *p* < 0.05, *p* < 0.005 vs. CTL cells, *p* < 0.05, *p* < 0.005 vs. TAD treated cells) at any interval time evaluated (24-48-72 h) (Figure S2B, *p* < 0.05, *p* < 0.005 vs. CTL cells, *p* < 0.05, *p* < 0.005 vs. TAD treated cells). Overall, these results strongly indicate that TAD promoted nuclear translocation of the AR and, also, facilitated AR transcriptional activity.

### 2.3. Tadalafil Increases In Vitro BCT-Mediated AR Downregulation and Cytostatic Effects on LNCaP Cells

To verify whether TAD was able to improve the pharmacological efficiency of BCT in treating hormone-sensitive PCa, cell viability was assessed in LNCaP cells treated with TAD (10^−6^ M), in the presence or absence of BCT (10^−4^ M). As shown in Figure 1D, as compared to BCT alone, cotreatment with TAD + BCT more efficiently reduced LNCaP proliferation after either 24 or 48 h incubation (TAD + BCT vs. BCT, *p* < 0.05). Although we did not detect any significant differences between BCT and BCT + TAD, we observed a trend of major reduction of AR protein expression in cells treated with TAD + BCT compared to BCT alone after 24 h (Figure 2C, BCT vs. CTL, *p* < 0.05; TAD + BCT vs. CTL, *p* < 0.005) and 48 h (Figure 2C, BCT vs. CTL, *p* < 0.005; TAD + BCT vs. CTL, *p* < 0.001). In addition, after 48 h the cotreatment induces a major reduction of AR protein expression in these treated cells as compared to cells treated only with BCT (Figure 2C, BCT vs. TAD, *p* < 0.05; TAD + BCT vs. TAD, *p* < 0.005).

### 2.4. Tadalafil Modulates Aromatase and Estrogen Receptor-ß Expression

**Figure 3:**
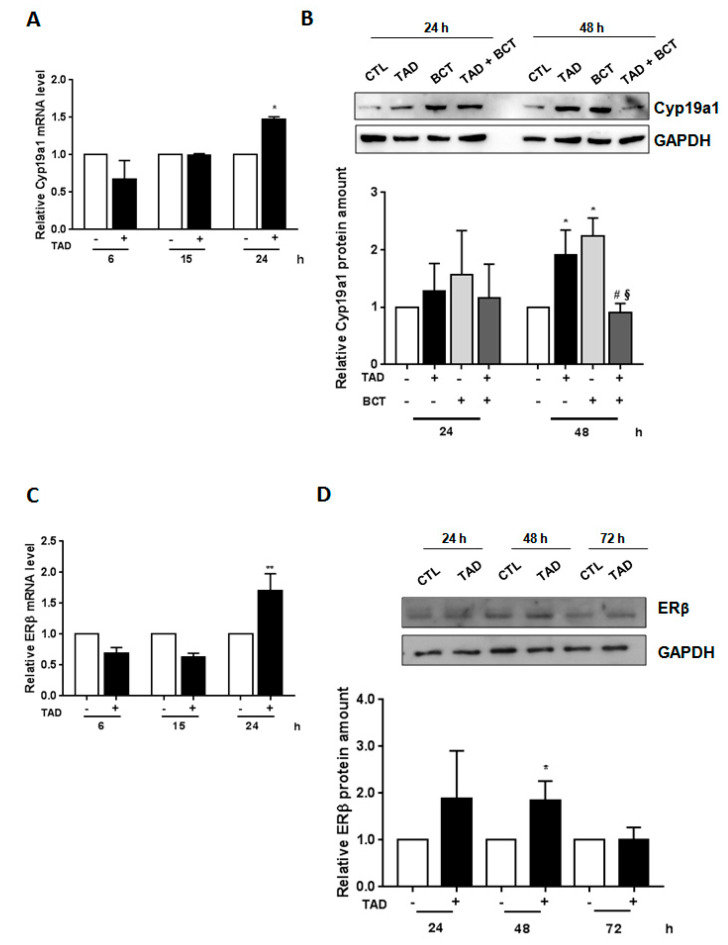
TAD dependent-increase of Cyp19a1 and ERβ expression and BCT-mediated Cyp19a1 downregulation. (**A**,**C**) Effect of TAD on Cyp19a1 and ERβ mRNA expression in LNCaP cells, either exposed or not to TAD (10^−6^ M) for 6, 15 and 24 h. (**B**) A representative Western blot of Cyp19a1 protein is shown in LNCaP cells cultured in the presence (+) or in the absence (−) of TAD (10^−6^ M) and/or BCT (10^−4^ M) for 24 and 48 h. (**D**) A representative Western blot of ERβ protein is shown in LNCaP cells cultured in the presence (+) or in the absence (−) of TAD (10^−6^ M) for 24, 48 and 72 h. GAPDH, control of protein loading. Results are shown as mean ± SE (*n* = 3) of three separate experiments. * *p* < 0.05, ** *p* < 0.005 vs. CTL cells, # *p* < 0.05 vs. TAD treated cells, § *p* < 0.05 vs. BCT treated cells.

Cyp19a1 converts androgens to estrogen [40] that in turn can impair AR expression [41]. Since we have recently shown that TAD modulates Cyp19a1 [31], we hypothesized that TAD could improve BCT-induced AR downregulation by promoting Cyp19a1 expression. Exposure of LNCaP cells to TAD (10^−6^ M) for 24 h upregulated Cyp19a1 mRNA expression (Figure 3A, TAD vs. CTL, *p*< 0.05). Moreover, as shown in Figure 3B, both TAD and BCT alone increased Cyp19a1 protein expression after 48 h (TAD vs. CTL, *p* < 0.05; BCT vs. CTL, *p* < 0.05). TAD + BCT cotreatment reduced Cyp19a1 protein expression after 48 h compared to either TAD (TAD + BCT vs. TAD, *p* < 0.05) or BCT alone (TAD + BCT vs. BCT, *p* < 0.05). These results indicate that the ability of TAD to improve BCT-induced AR downregulation might be mediated by Cyp19a1.

In addition, exposure of LNCaP cells to TAD (10^−6^ M) augmented estrogen receptor-beta (ERβ) levels. As shown in Figure 3C, a significant difference was observed in ERβ mRNA levels after 24 h of treatment between untreated and treated cells (Figure 3C, TAD+ vs. TAD−, *p* < 0.005). Moreover, TAD significantly increased ERβ protein expression (48 h, TAD vs. CTL, *p* < 0.05), which approximated to baseline levels after 72 h (Figure 3D).

## 3. Discussion

The results presented herein demonstrate for the first time that TAD can modulate AR expression in prostate cancer cells in vitro. We also show that TAD induced the stabilization and reduced the degradation of AR, which is more efficiently accumulated within the nucleus. Interestingly, this effect could lead to a potential anticancer action of TAD by enhancing the therapeutic effect of ADT.

**Figure 4:**
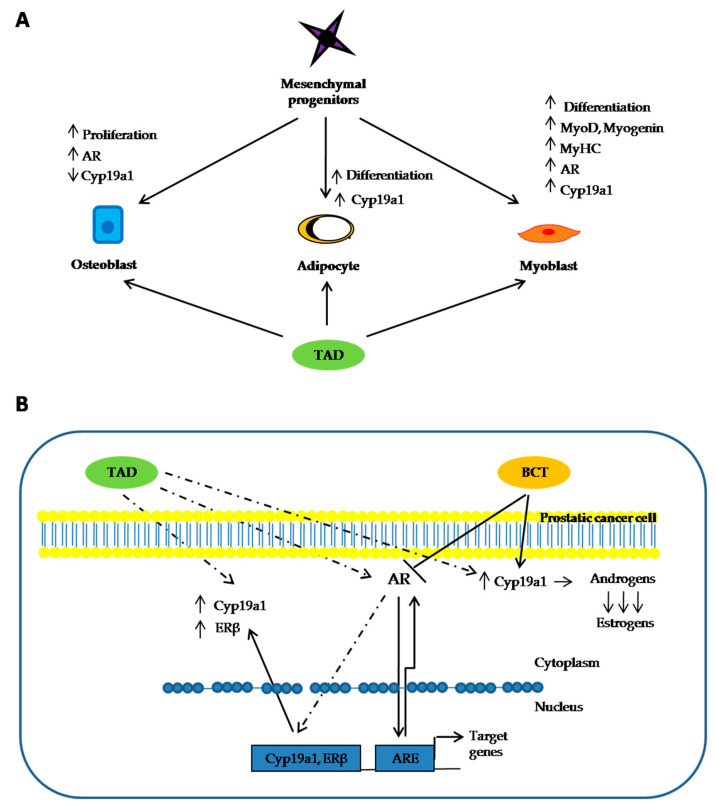
(**A**) Schematic representation of the effects of PDE5 inhibitor (PDE5i) tadalafil (TAD) on different cell lines on proliferation and differentiation. It is suggested that TAD may modulate the expression of molecules such as Cyp19a1 and the androgen receptor (AR). In particular in the osteoblastic cell, TAD leads to an increase of AR and a decrease of Cyp19a1 and it has been described an increase in cellular proliferation. In adipocyte cells TAD has a positive effect on Cyp19a1 expression. Moreover, in myoblasts, it has been reported to increase AR and Cyp19a1 proteins and of molecules involved in cell differentiation, such as MyoD, myogenin. (**B**) Molecular mechanism triggered by TAD in the prostate cancer cell line (dotted lines represent unexplained effects). Chronic exposure to TAD induced an increase in AR protein expression and an increase in gene and protein expression of ERβ and Cyp19a1. We hypothesize that chronic exposure to BCT may induce loss of ERβ due to induction of Cyp19a1, and cotreatment with TAD is able to block these effects.

PCa is a leading cause of death in adult males and often CRPC has a lower therapeutic response to conventional chemotherapy [42]. The expression of PDE5 and cGMP-signaling pathway in normal and cancerous prostate tissues and their possible involvement in carcinogenesis still remain controversial. TAD, sildenafil and vardenafil are employed to treat erectile dysfunction (ED) through the specific inhibition of cGMP-specific PDE5, responsible for cGMP degradation in the corpus cavernosum [43]. Currently, PDE5i are largely used as daily treatment for benign prostatic hyperplasia (BPH)-related lower urinary tract symptoms (LUTS) where they reduce spontaneous contractility of the glands, thereby reducing the muscle tone of the genitourinary tract [44] even if a specific inhibitory growth pattern on prostate tissue has not been clearly documented. Fibbi et al. reported PDE5 immunolocalization mainly in the fibromuscular stroma and vascular (endothelial and stem cells) in the rat and human prostate from BPH subjects [45]; by contrast, Ückert et al. immunolocalized PDE5 expression also at glandular and subglandular areas of human prostate cancer patients [46]. Zhang et al. reported an upregulation of PDE5 in hyperplastic human prostate thus providing a rationale for the high efficacy of PDE5 inhibitors for treating patients with LUTS/BPH with/without ED [47]. Although PDE5 inhibitors are largely used after oncological curative treatments for PCa, PDE5 immunolocalization studies in prostate adenocarcinomas have not been exhaustively reported in the literature. In order to properly localize PDE5 expression, Bisegna et al. [48] recently found PDE5 overexpression in the stromal compartment of hyperplastic prostate samples. Interestingly, their immunohistochemical study showed a 22% of PCa samples expressing PDE5 in the epithelial compartment compared to normal (8%) or hyperplastic samples (11%), and that such positivity was not correlated with the Gleason grading system. Recent data suggest that sildenafil and vardenafil may induce PDE5-independent apoptotic sensitization to doxorubicin (or other topoisomerase II inhibitors), thus suggesting a combinatory treatment as an important strategy for anti-CRPC development [49]. Given our previous finding of a Cyp19a1 and AR regulation by TAD in human adipocytes [31], osteoblast [32] and skeletal cell lines [33] (summarized in Figure 4A), respectively, we investigated potential actions of TAD in a different cellular model. In this PCa cell-line, the acute exposure of LNCaP to TAD did not affect cell viability and proliferation rate. TAD upregulates AR protein expression and transcriptional activity, without affecting neither metabolism nor proliferation of PCa cells. Several studies indicate that PCa is driven by the AR, a ligand-dependent transcription factor belonging to the nuclear receptor family. Multiple growth-promoting and survival pathways interact with AR signaling and are involved in PCa [50]. The rising prostatic specific antigen (PSA) levels measured in PCa patients, who become resistant to ADT, indicate that AR signaling remains an essential target for pharmacological intervention. Approaches with compounds interfering with AR signaling are currently being investigated in clinical studies [51]. Early clinical studies with agents target in programmed cell death protein 1 (PD-1) or programmed death-ligand 1 (PD-L1) are currently ongoing. As already demonstrated in other cell models [31,32,33], we confirm that TAD increases AR expression after 24 h exposure of LnCAP cell at the nuclear level; and promotes nuclear translocation of AR triggering its transcriptional activity. Additionally, cotreatment with AR-blocker BCT, produced a significant drop in LnCAP proliferation at 24 and 48 h, thus suggesting a putative add-on action of this drug in inhibiting AR-mediated PCa cell growth (see Figure 4B).

AR is the classical target for PCa prevention and treatment, but more recently estrogens and their receptors have also been implicated in both development and tumor progression. Increasing evidence demonstrate that local estrogen signaling mechanisms are required for prostate carcinogenesis and tumor progression [52]. The effects of aromatase inhibitors (AIs) on the human prostate due to systemic estrogen depletion are becoming clinically important due to their increasing use as an adjuvant therapy in postmenopausal women with breast cancer. Cyp19a1 converts androgens into estrogens, and the role of estrogens in the pathophysiology of PCa is not well established. Bonkhoff confirmed the hypothesis according to which estrogens may play a major role in the regulation of prostate growth in men [53]. In detail, estrogen ERβ is the most prevalent ER in the human prostate, while the estrogen receptor alpha (ERα) is restricted to basal cells of the prostatic epithelium and stromal cells. In high grade prostatic intraepithelial neoplasia (HGPIN), the ERα might be upregulated while a partial loss of the ERβ might occur, suggesting a potential action as tumor suppressor. The ERβ is generally retained in hormone naïve and metastatic prostate cancer, but it is partially lost in castration resistant disease [54]. The progressive emergence of the ERα and ERα-regulated genes (e.g., progesterone receptor, PS2, TMPRSS2-ERG fusion and NEAT1) during PCa progression and hormone refractory disease suggests that these tumors can bypass the AR by using estrogens and progestins for their growth. Indeed, in CRPC Cyp19a1 expression was significantly increased in tumor samples [55]. In PCa cell lines, the androgen receptor antagonist BCT increased Cyp19a1 expression and ERβ transcriptional activity. It is well known that steroids and drugs, which bind to the intracellular receptor, might have a bell-shape curve [56,57,58] upon the concentration of the molecule. Thus, we believe that the TAD plus BCT combination might exert a similar effect and, at high concentration, an inhibitory effect. Moreover, it has been shown that the combinatory use of two drugs, could either exert synergistic effects, or antagonistic actions, when the combination of two drugs leads to a smaller effect than expected [59]. In addition, gene expression signature predictive of CRPC with neuroendocrine differentiation revealed an over-representation of estrogen signaling [60]. Therefore, an increase in Cyp19a1expression might be one of the mechanisms underlying CRPC development.

Our study demonstrated for the first time that chronic exposure (48 h) to BCT produced a significant increase in Cyp19a1 mRNA in LnCAP cells, which was reverted by cotreatment with TAD. Indeed, anastrozole and selective AIs had been proposed for the treatment of men with advanced prostate cancer, but currently, results are still inconsistent [61]. Attia and Ederveen demonstrated that an increase of ERβ in prostate cells stimulates cell apoptosis and decreases cell cancer cell proliferation exerting a potential interesting pharmacological role in neoplastic lesions [62]. We herein speculate that the local increase in estrogen levels might activate ERβ intracellular pathway and that chronic exposure to BCT may induce loss of ERβ due to induction of Cyp19a1; the cotreatment with TAD is able to block these effects and thus one can hypothesize the maintenance of androgen responsiveness to antiandrogen therapy. In fact, TAD potentiated the antiproliferative activity of BCT in LNCaP cells.

The study has some limitations. We did not evaluate the potential effects of PDE5i treatment on cytokines (i.e., IFN-β) involved in the modulation of cell proliferation and differentiation of CRPC [63]. Furthermore, it was clear that the cotreatment of TAD and BCT strongly reverted the stimulation of Cyp19a1, but further studies aimed to clarify mechanism/s explaining enhancement of ADT by TAD are needed to evaluate the antitumor potential of these two molecules, and their role on the estrogen receptors function. Finally, we did not evaluate potential known interactions between androgen receptor blockade and downstream compounds, i.e., cAMP/PKA signaling, which may be involved in alternative pathways underlying castration-recurrent prostate cancer [64].

## 4. Materials and Methods

### 4.1. Reagents

Buffers and reagents were from Corning, and medium from PAN-Biotech. Charcoal stripped fetal bovine serum (FBS) and CHX were from Sigma Aldrich. TAD, BCT and MDV were purchased from Santa Cruz Biotechnology, dissolved in dimethylsulfoxide (Sigma Aldrich, St. Louis, MO, USA) and then added to cells at the concentrations indicated. Primary antibodies were as following: GAPDH, Millipore; tubulin, Abcam; AR, Cyp19a1, ERβ and Lamin A/C, Santa Cruz Biotechnology (Dallas, TX, USA). Trizol was purchased from Life Technologies, while the Cell Titer 96 Aqueous One Solution Cell Proliferation Assay kit was from Promega (Madison, WI, USA).

### 4.2. Cell Culture

LNCaP cells (a human androgen sensitive PCa cell line), DU-145 and PC3 (a human androgen insensitive PCa cell line) were cultured in RPMI 1640 medium, supplemented with 2 mM L-glutamine, 100 U/mL penicillin/streptomycin, 10% fetal bovine serum (FBS), at 37 °C, and 5% CO_2_ in a humidified incubator. AR-positive LNCaP cells and AR-negative DU-145 and PC3 were grown up to 70–80% confluence, starved overnight in serum-free medium, treated with TAD (10^−6^ M) or BCT (10^−4^ M) (or a combination of both), resuspended in medium w/o phenol red, supplemented with 10% charcoal stripped FBS (CS-FBS) and collected after short- (15 min) or long-term (24, 48 and 72 h) drug exposure. For the cotreatment with TAD and BCT, cells were first pretreated for 1 h with BCT (10^−4^ M) and then cotreated with TAD (10^−6^ M). In regards of the cotreatment with TAD and MDV, cells were first pretreated for 2 h with MDV (10^−5^ M) and then cotreated with TAD 10^−6^ M for 15 min. After overnight starvation in serum-free medium, cells were pretreated for 1hr with CHX, and then cotreated with TAD (10^−6^ M) for 3 h in medium w/o phenol red supplemented with 10% CS-FBS.

### 4.3. Cell Proliferation Assay

LNCaP cell viability was determined by the Cell Titer 96 Aqueous One Solution Cell Proliferation Assay kit, based on cell-mediated (3-(4,5-dimethylthiazol-2-yl)-5-(3-carboxymethoxyphenyl)-2-(4-sulfophenyl)-2H-tetrazolium (MTS) reduction to formazan, following the manufacturer’s protocol. Cells were plated at a density of 5000 cells/well in a 96-well culture plate, and cell viability was evaluated upon treatment with TAD (10^−6^ M) and BCT (10^−4^ M) for 24–48 h. The absorbance was measured at 490 nm using a plate reader (680 Microplate Reader; Bio-Rad, Hercules, CA, USA), and expressed as the optical density value (OD). Three independent experiments were performed, each in triplicate.

### 4.4. RNA Isolation and Quantitative Real-Time PCR

For RNA extraction, cells were washed twice with PBS 1X and lysed with Trizol according to the manufacturer’s instructions. The purity and integrity of total RNA was monitored by electrophoretic analysis through a denaturing agarose gel. Ultraviolet spectrophotometry (Eppendorf, Hamburg, DE) was used for RNA yield evaluation. Total RNA (1 µg) was treated with DNAse I Amplification Grade (Invitrogen, Life&Technologies, Carlsbad, CA, USA) and reverse-transcribed using the SuperScriptTM III (Invitrogen, Life&Technologies, Carlsbad, CA, USA). Quantitative real-time PCR was performed in Abi Prism 7500 light cycler (Applied Biosystem, Waltham, MA, USA), using power SYBR green PCR master mix (Applied Biosystem, Waltham, MA, USA) as indicated by the manufacturers. All primers were optimized for amplification checking the generation of a single amplicon in a melting curve assay and the efficiency in a standard curve amplification (>98% for each couple of primers).

Each analysis was performed in triplicate. Relative expression levels were calculated using the comparative cycle threshold (ΔΔCt) method, using cyclophilin A as an internal control. The sequences of the utilized primers were as follows:

- AR (NM_000044), FW: TAC CAG CTC ACC AAG CTC CT; REW: GAT GGG CTT GAC TTT CCC AG.
- Cyp19a1 (NM_000103), FW: ACT ACA ACC GGG TAT ATG GAGAA; REW: TCG AGA GCT GTA ATG ATT GTGC.
- ERβ (NM_001214902), FW: AGC ACG GCT CCA TAT ACA TACC; REW: TGG ACC ACT AAA GGA GAA AGGT
- Cyclophilin A (NM_021130), FW: GTC AAC CCC ACC GTG TTC TT; REW: AAA GTT TTC TGC TGT TTT TGGAAT C.
- PSA (NM_001030047), FW: GAGGAGTTCTTGACCCCAAAG; REW: CGCACACACGTCATTGGAAATA.

Each sample was always analyzed in duplicate in all the experiments performed.

### 4.5. Protein Extraction and Western Blot Analysis

After each treatment, cells were washed with phosphate-buffered saline (PBS) and lysed in fresh ice-cold RIPA buffer (150 mM NaCl, 50 mM Tris–HCl, pH 7.5, 500 μM EDTA, 100 μM EGTA, 1.0% Triton X-100 and 1% sodium deoxycholate) supplemented with a cocktail of protease and phosphatase inhibitors (Sigma Aldrich, St. Louis, MO, USA). Lysates were precleared by centrifugation and the resultant supernatant (total proteins) was collected and stored at −80 °C until use. The NE-PER nuclear and cytoplasmic extraction kit (Thermo Fisher Scientific, Waltham, MA, USA) was used to extract nuclear and cytoplasmic fraction of cell proteins.

The protein amount was measured using the Micro BCA Protein assay kit (Thermo Scientific, Waltham, MA, USA) and analyzed by Western blot (WB) analysis. An equal amount of protein extract (20 μg) was separated in SDS–PAGE (10–14%) and transferred to nitrocellulose membrane (GE Healthcare, Chicago, IL, USA). Membranes were saturated by soaking them at RT for 1 h with 5% (*w*/*v*) non-fat dry milk (Cell Signalling Technology, Danvers, MA, USA), in 20 mM Tris–HCl, 150 mM NaCl and 0.05% Tween 20. Then, they were incubated with specific antibodies overnight at 4 °C, washed three times with T-TBS pH 7.5, and subsequently incubated for 60 min at room temperature with HRP-labeled secondary antibody (Jackson Laboratories, Bar Harbor, ME, USA; diluition 1:10,000) in 5% non-fat dry milk, to detect the immunoreactive protein bands, which were visualized by enhanced chemiluminescence (GE Healthcare, Chicago, IL, USA). The bands were acquired on the ImageQuant LAS 4000 (GE Healthcare, Chicago, IL, USA) and quantified by ImageQuant TL Image analysis software (GE Healthcare, Chicago, IL, USA), using GAPDH, Lamin A/C and tubulin for normalization.

### 4.6. Luciferase Assay

Transfections were performed using the Lipofectamine 2000 reagent (Invitrogen), following the manufacturer’s protocol. The androgen-responsive synthetic reporter construct, ARE^4^LUC, was previously described [65,66]. Human rhabdomyosarcoma cell lines (RD) were used as a positive control [67]. Luciferase activity assay was performed following the manufacturer’s protocol (Promega).

### 4.7. Statistical Analysis

Statistical analysis was performed by using an unpaired Student’s *t*-test followed by a Mann Whitney post-hoc test (GraphPad Prism, San Diego, CA, USA). Data are presented as mean ± standard error (SE) of at least three independent experiments. *p* values of <0.05 were considered significant.

## 5. Conclusions

This study reported for the first time a novel action of TAD in a model of PCa cell line with respect to same actions previously described in other cell models in vitro [31,32,33]. The data herein presented suggest that TAD might lead to an increased response to antiandrogen compounds by increasing AR protein expression and activity in PCa cells in vitro. TAD treatment is widely used for the treatment of ED in patients unaware of bearing PCa. Whether these translational protective effects may apply to slow down the clinical progression of PCa to CRPC remains to be elucidated.

## Data Availability

The data presented in the study are available in the Supplementary data.

## Supplementary Materials

Supplementary materials can be found at https://www.mdpi.com/1422-0067/22/2/754/s1.

## Abbreviations

AR	Androgen Receptor
PCa	Prostate cancer
Cyp19a1	Aromatase
TAD	Tadalafil
LnCAP	Androgen-sensitive human PCa cell line
BCT	Bicalutamide
T	Testosterone
DHT	Dihydrotestosterone
AREs	Androgenr esponse elements
ADT	Androgen deprivation therapy
CRPC	Castration-resistant prostate cancer
cAMP	Cyclic adenosine monophosphate
cGMP	Cyclic guanosine monophosphate
PDE	Phosphodiesterase
PDE5i	PDE5 inhibitor
CHX	Cycloheximide
PSA	Prostatic specific antigen
MDV	MDV3100
CYT	Cytoplasmic fraction
NUC	Nuclear fraction
RD	Human Rhabdomyosarcoma Cell Lines
ED	Erectile dysfunction
BDH	Benign prostatic hyperplasia
ERβ	Estrogen receptor beta
LUTS	Lower urinary tract symptoms
PSA	Prostatic specific antigen
PD-1	Programmed cell death protein 1
PD-1L	Programmed death-ligand 1
AIs	Aromatase inhibitors
ERα	Estrogen receptor alpha
ERβ	Estrogen receptor beta
HGPIN	High grade prostatic intraepithelial neoplasia
