# Supplementary material for: Phosphodiesterase Type-5 Inhibitor Tadalafil Modulates Steroid Hormones Signaling in a Prostate Cancer Cell Line
Source: Int J Mol Sci. 2021 Jan 13;22(2):754. doi: 10.3390/ijms22020754 (PMC7828628; doi:10.3390/ijms22020754)
Supplement: Supplementary file 1 [file ijms-22-00754-s001.pdf]

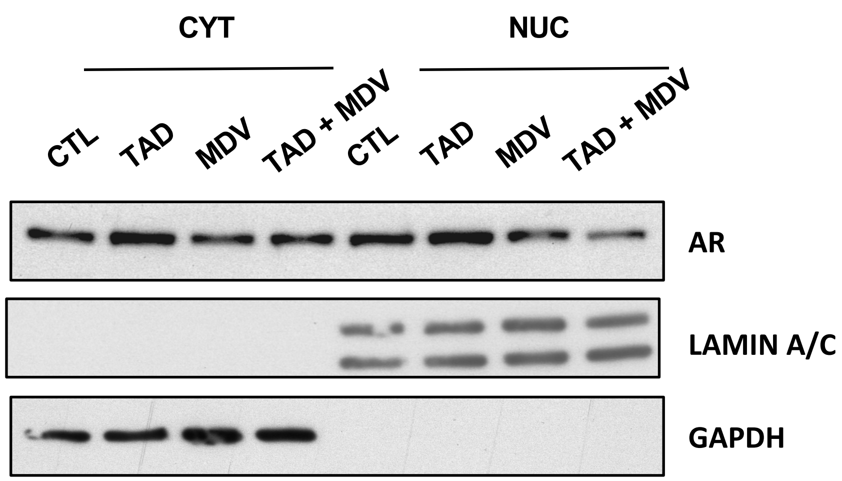

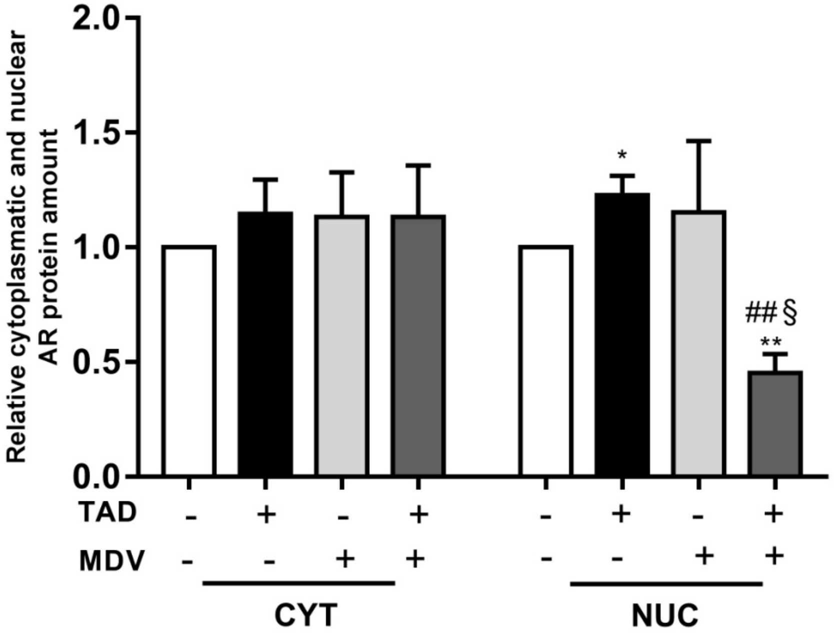

Figure S1. Effect of TAD on AR translocation. Representative Western blot analysis of AR protein expression in androgen-sensitive cell line LNCaP cultured in the presence (+) or in the absence (−) of TAD (10^{-6} M) and MDV3100 (MDV 10^{-5} M). Cells treated with MDV for 2 h by addition of TAD for 15 min. GAPDH and Lamin A/C were used as loading and purity controls of each cellular fraction. Results are represented as mean ± SE (n=3) of three independent experiments. * $p<0.05$, ** $p<0.005$ *vs* CTL cells, ## $p<0.005$ *vs* TAD treated cells, § $p<0.05$ *vs* MDV treated cells.

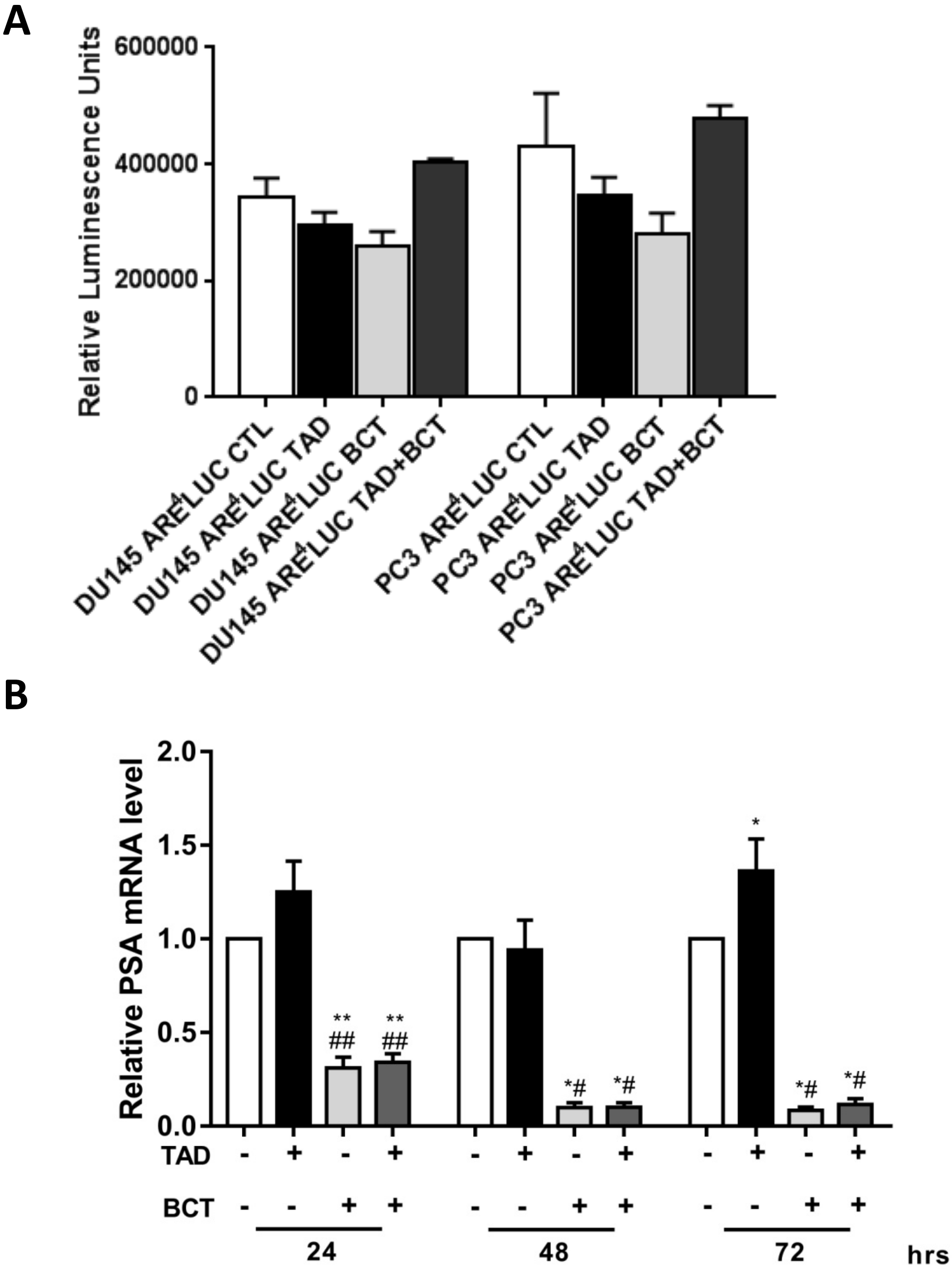

Figure S2. Effect of TAD on AR transcriptional activity in Prostatic cancer cells lines.(**A**) DU145 and PC3 were transfected with ARE^{4}LUC and treated in the presence (+) or in the absence (−) of TAD (10^{-6} M) and BCT (10^{-4}M). Luciferase activity was measured after 24 h. (**B**) Analysis of the AR-dependent genes PSA in LNCaP cells cultured treated in the presence (+) or in the absence (−) of TAD (10^{-6} M) and BCT (10^{-4}M). Results are represented as mean ± SE (n=3) of three independent experiments. * p<0.05, *** p<0.001*vs* CTL cells, # p<0.05, ## p<0.005, *vs* TAD treated cells treated cells.
